# Efficacy of bedaquiline in the treatment of drug-resistant tuberculosis: a systematic review and meta-analysis

**DOI:** 10.1186/s12879-021-06666-8

**Published:** 2021-09-17

**Authors:** Ming-Gui Wang, Shou-Quan Wu, Jian-Qing He

**Affiliations:** Department of Respiratory and Critical Care Medicine, West China Hospital, Sichuan University, No. 37, Guo Xue Alley, Chengdu, Sichuan People’s Republic of China

**Keywords:** Bedaquiline, Tuberculosis, Multidrug resistance, Extensively drug resistant

## Abstract

**Background:**

Drug-resistant tuberculosis (DR-TB) remains a major public health concern worldwide. Bedaquiline, a novel diarylquinoline, was added to the WHO-recommended all-oral regimen for patients with multidrug-resistant tuberculosis. We performed a systematic review and meta-analysis to determine the effect of bedaquiline on tuberculosis treatment outcomes.

**Methods:**

We searched the PubMed, Web of Science and EMBASE databases for relevant studies published up to March 12, 2021. We included studies in which some participants received bedaquiline and others did not. Stata version 16.0 (Stata Corp., College Station, Texas, USA) was used to analyze the results of the meta-analysis. Risk ratios (RRs) with 95% confidence intervals (95% CIs) were calculated to evaluate the effect of bedaquiline on drug-resistant tuberculosis. Between-study heterogeneity was examined by the I-squared test. Randomized controlled trials were assessed for quality using the Jadad scale, and cohort studies were assessed using the Newcastle–Ottawa scale.

**Results:**

Eight studies, including 2 randomized controlled trials and 6 cohort studies involving a total of 21,836 subjects, were included. When compared with the control, bedaquiline treatment was associated with higher rates of culture conversion (risk ratio (RR):1.272 (1.165–1.389), P < 0.001). We found substantial evidence of a significant reduction in all-cause death (RR: 0.529 (0.454–0.616), P < 0.001)) in the bedaquiline treatment group. There was no significant reduction in treatment success (RR = 0.980 (0.948–1.013, P = 0.234)).

**Conclusions:**

This study demonstrated that compared with patients who do not receive bedaquiline, this drug has the potential to achieve a higher culture conversion rate and a lower mortality risk among drug-resistant tuberculosis cases.

**Supplementary Information:**

The online version contains supplementary material available at 10.1186/s12879-021-06666-8.

## Background

Tuberculosis (TB) remains an important global infectious disease. TB is caused by mycobacterium tuberculosis (MTB) and remains one of the leading causes of infection-related death worldwide. According to the World Health Organization (WHO), there were 10.0 million (range, 8.9–11.0 million) new TB patients in 2019 [1]. Globally, an estimated 1.4 million TB deaths occurred in 2019, including 1.2 million among human immunodeficiency virus (HIV)-negative people and an additional 208,000 deaths among HIV-positive people [1]. Drug-resistant TB (DR-TB) is a major public health concern. Rifampicin-resistant TB (RR-TB) requires treatment with second-line drugs. Multidrug-resistant TB (MDR-TB) is resistant to both rifampicin and isoniazid (the two most effective anti-TB drugs), and extensively drug-resistant tuberculosis (XDR-TB) is MDR-TB that is also resistant to fluoroquinolone, and injectable agent. Globally, in 2019, 3.3% of new cases and 18% of previously treated cases had MDR/RR-TB. It is estimated that there were 465 000 incident cases of MDR/RR-TB in 2019, and the global proportion of RR-TB cases estimated to have MDR-TB was 78% [1]. The three countries with the heaviest burden of drug-resistant tuberculosis are India, China and the Russian Federation [1].

In 2019, total of 177,099 MDR/RR-TB patients were reported to have received treatment [1], up from 156,205 in 2018. However, 86% of the 206,030 people with MDR/RR-TB who were detected and notified in 2019 started MDR-TB treatment. Treatment outcomes for MDR/RR-TB remain poor even in advanced healthcare systems. Overall, only 57% of MDR/RR-TB patients in the 2017 cohort successfully completed treatment (cured or treatment completed) [1]. Hence, unsuccessful treatment of MDR-TB is a key problem that requires action.

The novel diarylquinoline, bedaquiline, was added to the WHO-recommended all-oral regimen to replace the injectable treatments for MDR-TB patients [2]. Bedaquiline has been shown to improve sputum conversion rates in clinical studies [3, 4] and was shown to improve treatment outcomes in some observational studies [5–7]. It is thus necessary to review and summarize the overall treatment outcomes for MDR-TB patients who were treated with bedaquiline in recent years. We conducted this systematic review and meta-analysis to summarize the existing evidence of the effect of bedaquiline on DR-TB treatment outcomes.

## Methods

The meta-analysis was prepared based on the Preferred Reporting Items for Systematic Reviews and Meta-Analyses guidelines for systematic reviews and meta-analyses [8]. Since this was a meta-analysis of existing articles and no individual patient data were handled, ethical approval was unnecessary for this study.

### Search strategy and study selection

The PubMed, Web of Science and EMBASE databases was searched to identify relevant studies (Additional file 1: Table S1). English-language studies published until March 12, 2021, were retrieved using the following keywords: “bedaquiline”, or “tuberculosis,” or “multidrug resistant tuberculosis” or “extensively drug resistant tuberculosis”, and their synonyms or similar words. Two independent reviewers (MG and SQ) read and assessed the titles and abstracts of all articles identified by the search strategy. The full-text study reports of all potentially eligible studies were also independently screened by two review authors (MG and SQ) according to a standardized form containing the inclusion and exclusion criteria.

The inclusion criteria were: (1) patients were aged ≥ 18 years; (2) had laboratory-confirmed DR-TB; (3) and received anti-TB therapy containing bedaquiline as an intervention; (4) the control group was treated with drugs other than bedaquiline; (5) culture conversion or outcomes of success (including cure or treatment completion), failure, and death according to the WHO classification were reported [9]; and (6) the study was designed as a retrospective study, randomized controlled trial, or prospective cohort study. When data were duplicated or reported in more than one study, the first published study was included in the meta-analysis.

Articles were excluded if they were editorials, case reports, conference abstracts, animal studies, or had a sample size of less than 10.

### Assessment of methodological quality

All studies included in the meta-analysis were independently assessed for quality by 2 reviewers, and the high-quality studies were further analyzed. For randomized controlled trials (RCTs), the two review authors independently used the Jadad scale [10] to assess the methodological quality of each included study by using the following variables: random scheme and allocation concealment, blinding of participants, and follow-up. The maximum score was five points. A score of ≥ 3 was considered to indicate high quality. For cohort studies, the quality of studies was assessed with a modified version of the Newcastle–Ottawa scale (NOS) (http://www.ohri.ca/programs/clinical_epidemiology/oxford.asp) by two reviewers independently. Studies were evaluated on the basis of adequate participant selection, comparability of studies based on design and analysis, and adequate ascertainment of outcomes. This scale awards a maximum of nine points. A score of > 7 was considered to indicate high quality.

### Data extraction

Two review authors (MG and SQ) worked independently to extract data on the following characteristics: study characteristics (author; publication year; country, study design), characteristics of participants (sample size, gender, age, HIV coinfection), intervention arms and controls (intervention drug and dose, follow-up duration, and anti-TB therapy protocol), and treatment outcomes (culture conversion, treatment success (cure or treatment completed), and death). Disagreements were resolved through discussion and consensus.

### Statistical analysis

All the statistical analyses were performed by using the Stata version 16.0 (Stata Corp., College Station, Texas, USA). To evaluate the effect of bedaquiline on drug-resistant tuberculosis, meta-analysis calculations were performed using individual data from patients with clear treatment outcomes (culture conversion and treatment success (including cure or treatment completion) and all-cause mortality). The risk ratio (RR) and 95% confidence interval (95% CI) was used as the measure of treatment outcome (all-cause mortality, culture conversion, or treatment success). Between-study heterogeneity was examined by the I-squared test [11]. Publication bias was tested by Egger’s linear regression test and Begg’s test.

## Results

### Study flow diagram

**Fig. 1:**
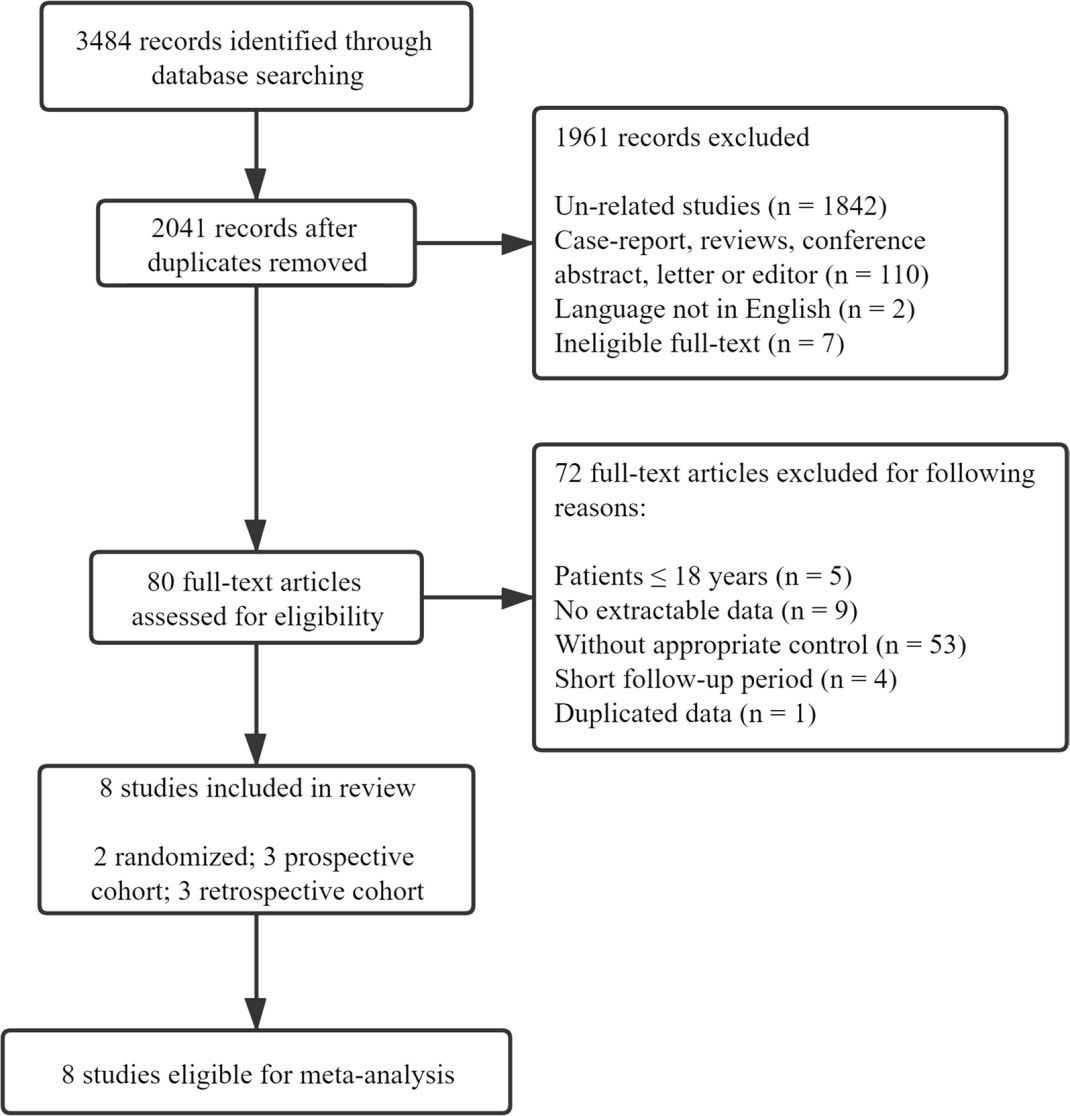
Study flow diagram

A total of 3484 citations were identified from the scientific literature search. After duplicates were removed, the title and abstract of 2041 records were screened, and 80 articles were found to be relevant for full-text analysis and reference list screening. From these, 72 articles did not fulfil the inclusion criteria and were excluded, and 8 studies were identified as eligible for inclusion in the meta-analysis [3–7, 12–14] (Fig. 1).

### Characteristics of included studies

The characteristics of the studies and the number of cases analyzed in the systematic review and meta-analysis are summarized in Table 1. The 8 included studies were conducted in 13 countries. Regarding regional distribution, more than half were conducted in South Africa [3–7, 14] (Table 1). Approximately 75% (n = 6) of the studies were published in the last 5 years. Of the eight included studies, two were RCTs [3, 4], three were retrospective cohort studies, and three were prospective cohort studies [5–7, 12–14].

**Table 1:** Characteristics of studies included in the meta-analysis

Study	Year	Study design	Country	Recruitment dates	Age (years)	Males (%)	HIV-positive (%)	Follow‐up	Duration	Bedaquiline-usement	Background regimen	Drug resistance	Bedaquiline treatment	Control	Total
Diacon et al.	2014	Randomized controlled trial	Brazil, India, Latvia, Peru, the Philippines, Russia, South Africa, and Thailand	NA	34 (18–63)	85 (63.39)	19 (14.39)	At 8, 24 and 72 weeks	120-week	400 mg once daily for 2 weeks, followed by 200 mg three times a week for 22 weeks	Ethionamide, pyrazinamide, ofloxacin, kanamycin, and cycloserine	MDR-TB	66	66	132
Kurbatova et al.	2015	Prospective cohort study	Philippines, South Africa, Peru, Russia, South Korea, Latvia, Thailand, Taiwan, and Estonia	January 1, 2005–December 31, 2008	> 18	613 (48.88)	159 (12.68)	Monthly	> 18 months	NA	Based on WHO and local treatment guidelines	MDR-TB	302	952	1254
Kim et al.	2018	Retrospective cohort	Korea	January 2015 and October 2017	52 (40.5–60)	49 (80.33)		Monthly	> 6 months	> 1 month (210 to 237 days)	Based on WHO	MDR-TB	50	11	61
Kempker et al.	2020	Prospective cohort study	Tbilisi, Georgia	December 2015 to May 2017	≥ 16	78 (82.11)	2 (2.11)	Monthly	20–24 months	171 (166–190) days	Linezolid, cycloserine, clofazimine, and an injectable agent. Delamanid-based regimen in control	MDR-TB	64	31	95
Schnippel et al.	2018	Retrospective cohort	South African	July 1, 2014, to March 31, 2016	36 (29–44)	10,959 (55.86)	13,893 (70.82)	Every 2 weeks for the first month, then monthly for 5 months	> 18 months	24 weeks	Kanamycin, moxifloxacin, ethionamide, terizidone, and pyrazinamide	RR-TB, MDR-TB, XDR-TB	1016	18,601	19,617
Zhao et al.	2019	Retrospective cohort	South African	October 2014 to October 2016	> 18	190 (57.58)	233 (70.61)	Monthly	12 months	400 mg once daily for 2 weeks, followed by 200 mg three times a week for 22 weeks	Moxifloxacin, pyrazinamide, ethionamide, high-dose isoniazid, ethambutol, and terizidone	MDR-TB	162	168	330
Olayanju et al.	2018	Prospective cohort study	South African	January 2008 and June 2017	> 18	161 (59.19)	134 (49.26)	Monthly	24 months	NA	Para-aminosalicylic acid, clofazimine, capreomycin and second-/fourth-generation fluoroquinolones	XDR-TB	68	204	272
Dooley et al.	2021	Randomized controlled trial	South African and Peru	Aug 26, 2016, and July 13, 2018	34 (20–49)	63 (75.00)	31 (36.90)	Every 2 weeks until week 24, then at week 28	> 7 months	400 mg once daily for 2 weeks, followed by 200 mg three times a week for 22 weeks	Capreomycin, cycloserine, ethambutol, ethionamide, pyrazinamide, levofloxacin, isoniazid, terizidone, Linezolid. Delamanid in control	MDR-TB and RR-TB	56	28	84

In total, there were 21,845 patients from the 8 included studies (Table 1), including 1784 patients treated with bedaquiline and 20,061 not treated with bedaquiline. Nearly 66.3% were HIV positive, and 55.9% were males. The antiviral treatment of HIV-positive patients in both the case group and the control group was consistent among the studies. In the bedaquiline treatment group, bedaquiline was generally administered at 400 mg daily for 2 weeks, followed by 200 mg three times per week for 22 weeks. The duration of treatment was > 6 months. The sample sizes of the studies included in the meta-analysis ranged from 61 [12] to 19,617 [7].

### Treatment outcomes

**Fig. 2:**
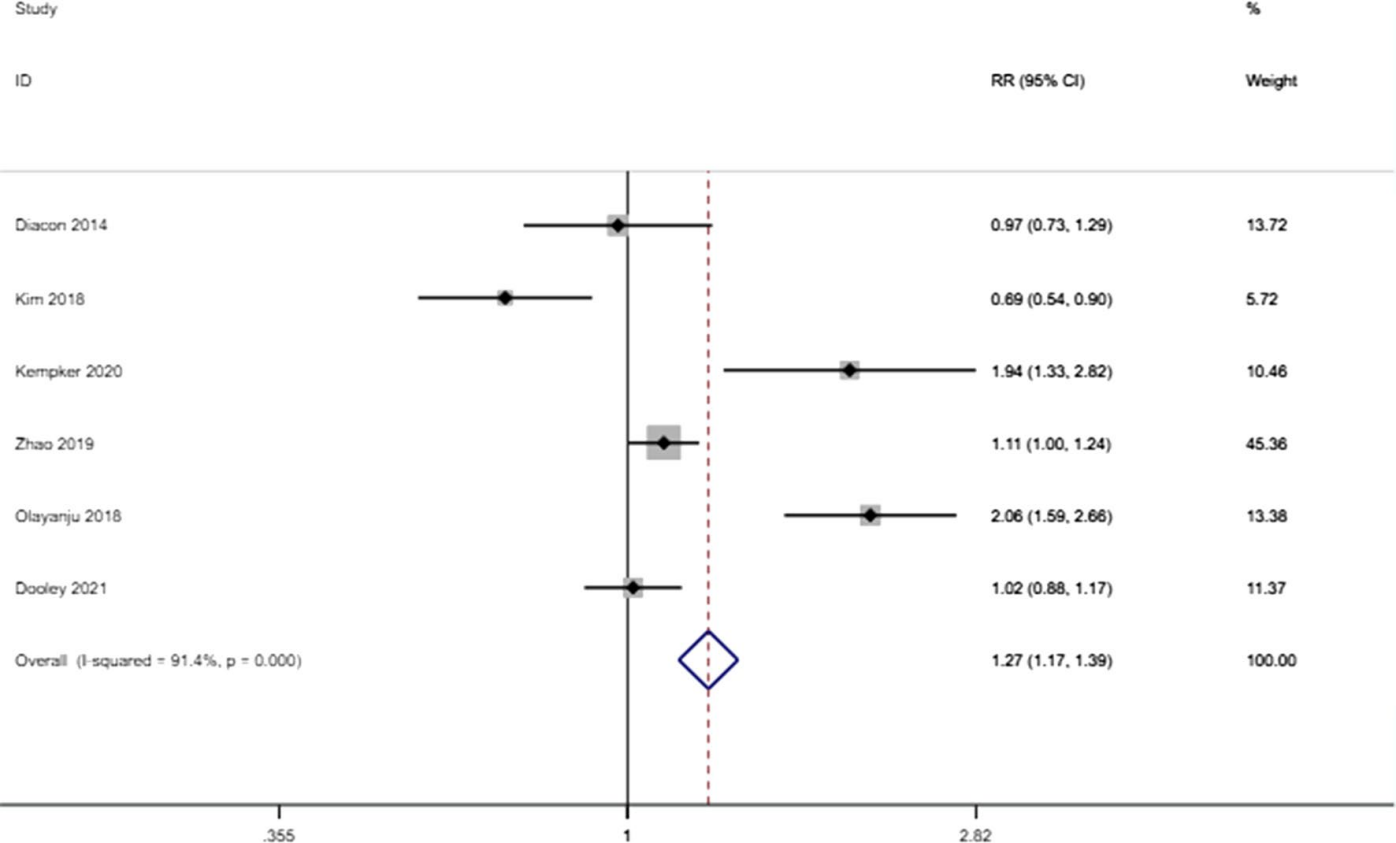
Forest plot of the effect of bedaquiline on culture conversion. *RR* risk ratio, *CI* confidence interval

**Fig. 3:**
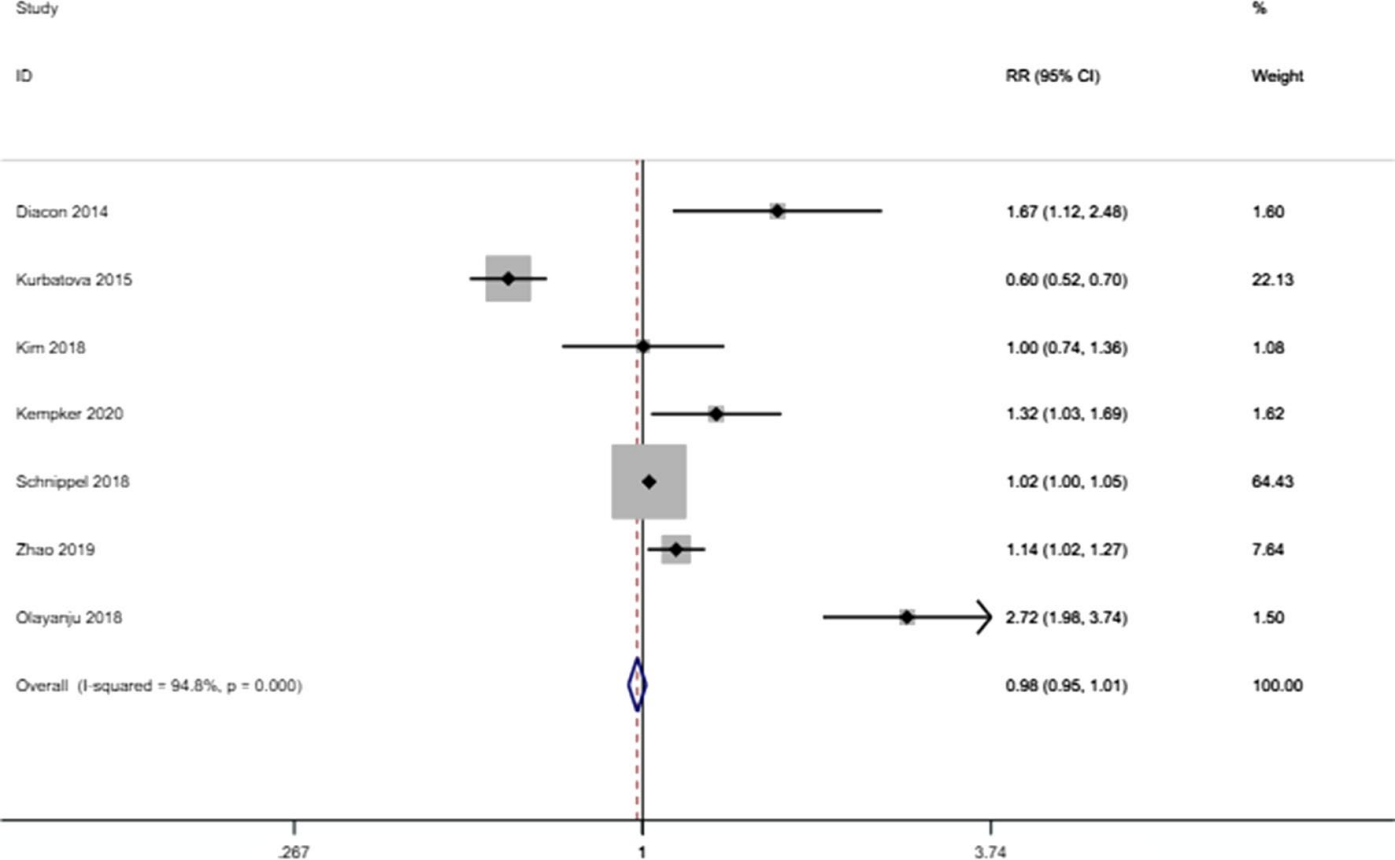
Forest plot of the effect of bedaquiline on treatment success. *RR* risk ratio, *CI* confidence interval

**Fig. 4:**
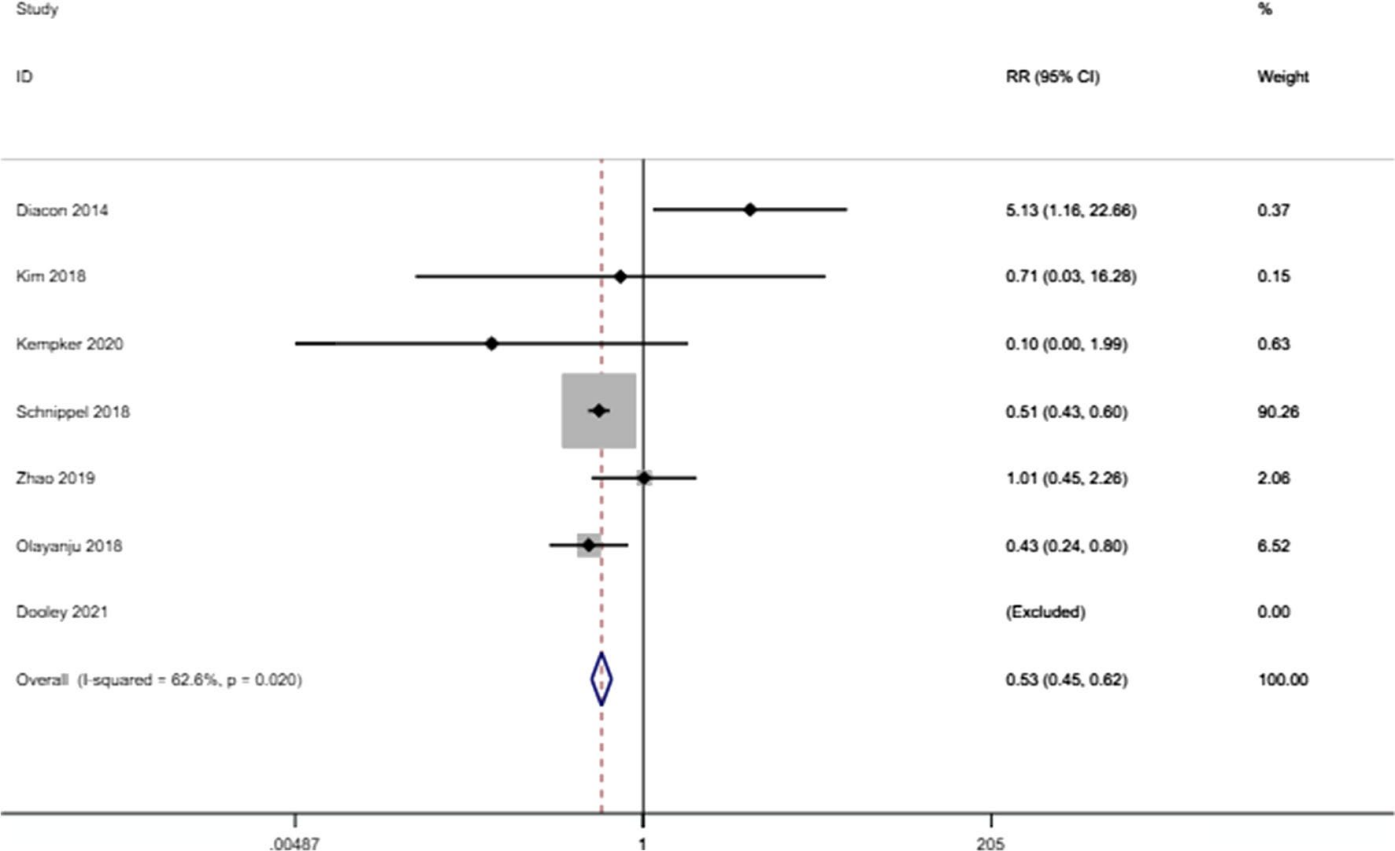
Forest plot of the effect of bedaquiline on all-cause mortality. *RR* risk ratio, *CI* confidence interval

The meta-analysis found that the risk of culture conversion was higher in patients receiving bedaquiline-containing regimens than in those not receiving bedaquiline-containing regimens (RR: 1.272 (1.165–1.389), P < 0.001) (Fig. 2). However, bedaquiline treatment did not have a statistically significant effect on the outcome of success (RR: 0.980 (0.948–1.013), P = 0.234) (Fig. 3). There were significant differences in the proportion of deaths due to any cause between those who received bedaquiline-containing regimens versus the controls. Patients receiving bedaquiline had a lower risk of all-cause mortality than those not receiving bedaquiline (RR: 0.529 (0.454–0.616), P < 0.001) (Fig. 4).

Significant heterogeneity was detected between the results of the studies, with an I^2^ value of 91.4% for culture conversion, 94.8% for successful treatment, and 62.6% for all-cause mortality. Due to the significant heterogeneity, we performed sensitivity analyses to explore the sources of heterogeneity. The heterogeneity was significantly reduced after the removal of Diacon (2014) (from 62.6% to 6.2%) for all-cause mortality [4].

### Assessment of risk of bias and publication bias

We assessed the risk of bias for the included RCTs using the Jadad scale, and the two included RCTs were of high quality (score ≥ 3). For cohort studies, we assessed the risk of bias using the NOS tool, and all included cohort studies were considered to be high quality. The results of the risk of bias analysis for the included studies are summarized in Additional file 1: Tables S2 and S3.

**Fig. 5:**
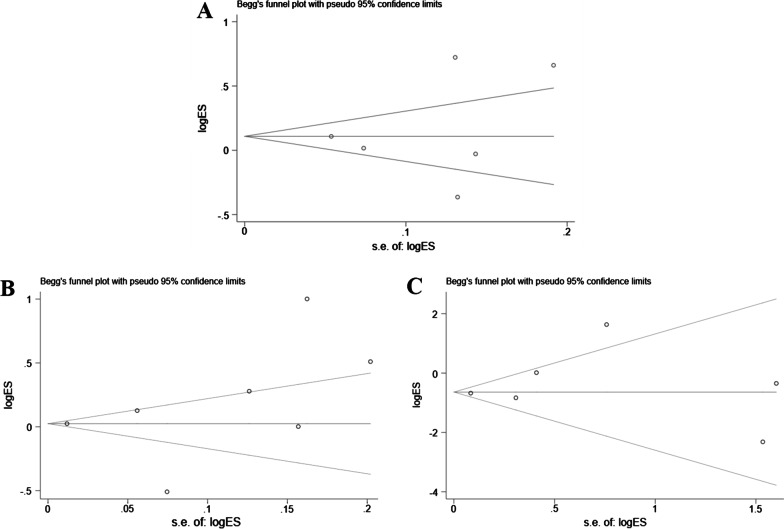
Funnel plots for publication bias. **A** Funnel plots for publication bias of culture conversion; **B** Funnel plots for publication bias of treatment success; **C** Funnel plots for publication bias of all-cause death. *logor* natural log of odds ratio, *s.e. of logor* standard error of logor

Begg’s and Egger’s regression tests were performed to assess publication bias. No substantial publication bias was found by either test. The Begg’s funnel plot is shown in Fig. 5.

## Discussion

To our knowledge, this is the first meta-analysis to investigate the effects of bedaquiline on patients with DR-TB. We analyzed data from 8 studies conducted in 13 countries, including 21,836 DR-TB patients. The results of this meta-analysis revealed the efficacy of bedaquiline in the treatment of drug-resistant tuberculosis.

For DR-TB, especially MDR/RR-TB and XDR-TB, bedaquiline was always administered in combination with other antitubercular drugs. Thus, treatment outcomes may not be entirely attributable to bedaquiline. Nevertheless, since all patients with DR-TB were treated with a background regimen, we believe that bedaquiline may be the most important factor affecting the treatment outcome in this meta-analysis. We found that bedaquiline could increase culture conversion (RR: 1.272 (1.165–1.389), P < 0.001) and decrease the risk of all-cause mortality (RR: 0.529 (0.454–0.616), P < 0.001). However, the administration of bedaquiline did not increase treatment success among DR-TB patients (P = 0.234).

Bedaquiline is a new antituberculosis drug belonging to the diarylquinoline class of compounds. It contains a quinolinic central heterocyclic nucleus with alcohol and amine side chains that play an important role in antituberculosis activity [15]. Studies have shown that bedaquiline is an inhibitor of mycobacterial ATP synthase; it binds to and perturbs the a-c subunit interface of Fo and leads to an ineffective proton cycle, which is fatal to mycobacterium [16, 17]. A multicenter study conducted in 25 centers and 15 countries on five continents found that at the end of treatment, the negative sputum smear and culture conversion rates in MDR-TB cases were 88.7% and 91.2%, respectively, and 71.3% achieved treatment success [18]. In other words, bedaquiline-containing regimens achieved high conversion and success rates when used to treat MDR-TB patients [18]. Another retrospective French cohort study showed that 97% of culture-positive TB patients achieved culture conversion after 6 months of bedaquiline treatment [19]. Our study evaluated the efficacy of bedaquiline for the treatment of DR-TB in RCTs and cohort studies. We found that DR-TB patients can benefit from the use of bedaquiline; such treatment can achieve a better sputum conversion rate and a lower risk of death. The WHO consolidated guidelines on DR-TB treatment recommend bedaquiline as one of the priority drugs (group A) for MDR-TB patients [2]. The use of bedaquiline may constitute a new era in the treatment of DR-TB patients, contributing to curbing the spread of this disease and reducing its mortality.

Taune et al. conducted a retrospective cohort study to describe the implementation of bedaquiline treatment and assess the safety and interim effectiveness for MDR-TB patients commenced on bedaquiline. The results showed that bedaquiline is a safe and well-tolerated drug with good interim effectiveness [20]. Studies of children and adolescents with DR-TB also show that bedaquiline-containing regimens are effective and well tolerated in children and adolescents, which may provide new directions for tuberculosis treatment in this group and contribute to the global strategy to end tuberculosis [20–22]. However, we did not evaluate the efficacy of bedaquiline in the treatment of child and adolescent patients with DR-TB, and further studies are needed.

There are many adverse reactions to bedaquiline, such as hyperuricemia, nausea, arthralgia, liver injury and QT prolongation [18–24]. Guglielmetti et al. found that nearly 20% of patients experienced a >  = 60-ms increase in QT interval, leading to bedaquiline discontinuation in 6% of patients [19]. A multicenter study found that adverse events presumably due to bedaquiline occurred in 19.4% of treated patients, and 5.8% of patients interrupted their bedaquiline treatment because of adverse events [18]. It is thought that most patients treated with bedaquiline will have adverse drug reactions, but most reactions are mild and do not lead to discontinuance [18, 19, 23, 25]. However, fatal arrhythmias can cause death [23].

Our review has some limitations. First, we included cohort studies and RCTs, which may have led to heterogeneity. Second, due to the limited data, we were unable to evaluate the safety of bedaquiline in the treatment of MDR/RR-TB and XDR-TB, and further studies are needed. In addition, we did not evaluate the effect of bedaquiline on DR-TB treatment outcomes among HIV-positive, child or adolescent patients. Further research focusing on these populations is necessary. Third, only eight studies were included in this meta-analysis, and the sample size of some of these studies was small. Additional randomized controlled trials with larger sample sizes are needed to further evaluate the efficacy and safety of bedaquiline in the treatment of DR-TB. Finally, our review processes had some limitations. To ensure feasibility, we were only able to include published articles, and unpublished articles were not screened. Furthermore, the language was limited to English, and articles published in other languages were not reviewed.

## Conclusion

The use of bedaquiline combined with other active drugs has the potential to achieve a higher culture conversion rate and a lower mortality risk among MDR/RR-TB and XDR-TB patients compared with those who do not receive this drug. Thus, the use of bedaquiline in DR-TB patients should be encouraged.

## Supplementary Information

**Additional file 1: Table S1.** Search strategy. **Table S2.** The Jadad scale of randomized controlled trials. **Table S3.** The Newcastle-Ottawa quality assessment scale of cohort studies.

## Data Availability

The datasets used and/or analysed during the current study are available from the corresponding author on reasonable request.

## Abbreviations

TB: Tuberculosis
MTB: Mycobacterium tuberculosis
WHO: World Health Organization
HIV: Human immunodeficiency virus
DR-TB: Drug-resistant tuberculosis
RR-TB: Rifampicin-resistant TB
MDR-TB: Multidrug-resistant TB
XDR-TB: Extensively drug-resistant tuberculosis
RCT: Randomized controlled trial
NOS: Newcastle–Ottawa Assessment Scale
RR: Risk ratio
CI: Confidence interval
