# Supplementary material for: Efficacy of bedaquiline in the treatment of drug-resistant tuberculosis: a systematic review and meta-analysis
Source: BMC Infect Dis. 2021 Sep 17;21:970. doi: 10.1186/s12879-021-06666-8 (PMC8447831; doi:10.1186/s12879-021-06666-8)
Supplement: Supplementary file 1 — Additional file 1: Table S1. Search strategy. Table S2. The Jadad scale of randomized controlled trials. Table S3. The Newcastle-Ottawa quality assessment scale of cohort studies. [file 12879_2021_6666_MOESM1_ESM.doc]

Table S1: Search strategy.

| **Databases** | **Search strategy** |
| --- | --- |
| **Medline** | 1.Tuberculosis, Multidrug‐Resistant"[Mesh] OR "Extensively Drug‐Resistant Tuberculosis"[Mesh] |
|  | 2. DR‐TB OR MDR‐TB OR XDR‐TB ab, kw, ti. |
|  | 3.Tuberculosis OR TB ab, kw, ti. |
|  | 4.Bedaquiline ab, kw, ti. |
|  | 5.“Bedaquiline” [Mesh]. |
|  | 6. 1 OR 2 OR 3 |
|  | 7.4 OR 5 |
|  | 8.6 AND 7 |
| **Web of science** | 1.TS = (tuberculosis OR TB OR multi-drug resistant tuberculosis OR MDR-TB OR drug-resistant tuberculosis OR DR-TB OR Extensively Drug‐Resistant Tuberculosis OR XDR-TB) |
|  | 2.TI = (tuberculosis OR TB OR multi-drug resistant tuberculosis OR MDR-TB OR drug resistant tuberculosis OR DR-TB OR Extensively drug-resistant tuberculosis OR XDR-TB) OR AB = (tuberculosis OR TB OR multi-drug resistant tuberculosis OR MDR-TB OR drug resistant tuberculosis OR DR-TB OR Extensively drug-resistant tuberculosis OR XDR-TB) OR AK = (tuberculosis OR TB OR multi-drug resistant tuberculosis OR MDR-TB OR drug resistant tuberculosis OR DR-TB OR Extensively drug-resistant tuberculosis OR XDR-TB) |
|  | 3.TS = (bedaquiline) OR TI = (bedaquiline) OR AB = (bedaquiline) OR AK = (beadquiline) |
|  | 4. 1 OR 2 |
|  | 5.3 and 4 |
| **Embase** | 1. Multidrug resistant tuberculosis [Emtree] OR “extensively drug resistant tuberculosis” [Emtree] OR “drug resistant tuberculosis” [Emtree] |
|  | 2. DR‐TB OR MDR‐TB OR XDR‐TB ab, kw, ti. |
|  | 3. Tuberculosis OR TB ab, kw, ti. |
|  | 4. “Bedaquiline” [Emtree]. |
|  | 5. Bedaquiline ab, kw, ti. |
|  | 6. 1 OR 2 OR 3 |
|  | 7. 4 OR 5 |
|  | 8. 6 AND 7 |

Table S2: The Jadad scale of randomized controlled trials.

| Study | Randomization | | Double blinding | | Withdrawals and dropouts | Total |
| --- | --- | --- | --- | --- | --- | --- |
|  | Mention "random" | The method of generating randomization sequence is suitable | Double blind | The blind method is suitable |  |  |
| Diacon 2014 | 1 | 0 | 1 | 1 | 1 | 4 |
| Dooley 2021 | 1 | 1 | 0 | 0 | 1 | 3 |

Table S3: The Newcastle-Ottawa quality assessment scale of cohort studies

| Study | Selection | | | | Comparability | | Assessment of outcome | | | Total |
| --- | --- | --- | --- | --- | --- | --- | --- | --- | --- | --- |
|  | Representativeness of the exposed cohort | Selection of the non exposed cohort | Ascertainment of exposure | Outcome not present at start of study | study controls for the most important factor DST | Studies controlling the other main factors age | Assessment of outcome with independency | Adequacy of follow up length | Lost to follow up acceptable (less than 20%) |  |
| Kurbatova 2015 | * | * | * | * | * |  | * | * | * | 8 |
| Kim 2018 | * | * | * | * | * | * | * | * | * | 9 |
| Kempker 2020 | * | * | * | * | * | * | * | * | * | 9 |
| Schnippel 2018 | * | * | * | * | * |  | * | * | * | 8 |
| Zhao 2019 | * | * | * | * | * | * | * | * | * | 9 |
| Olayanju 2018 | * | * | * | * | * | * | * | * | * | 9 |
